# Are equine-assisted services beneficial for military veterans with post-traumatic stress disorder? A systematic review and meta-analysis

**DOI:** 10.1186/s12888-024-05984-w

**Published:** 2024-08-01

**Authors:** Maisy Provan, Zubair Ahmed, Andrew R. Stevens, Amanda V. Sardeli

**Affiliations:** 1 Institute for Inflammation and Ageing, College of Medical and Dental Sciences, University of Birmingham, Edgbaston, Birmingham, B15 2TT UK; 2 Centre for Trauma Sciences Research, University of Birmingham, Edgbaston, Birmingham, B15 2TT UK

**Keywords:** Military veterans, PTSD, Equine-assisted services, Systematic review, Peer support

## Abstract

**Background:**

Equine-assisted services (EAS) involves the use of horses within therapy, learning or horsemanship sessions and has been used with military veterans suffering from post-traumatic stress disorder (PTSD). This study systematically reviewed existing research on the use of EAS in the treatment of PTSD in military veterans and evaluated its effectiveness.

**Methods:**

A systematic review was performed, in May 2023, with searches and data extraction carried out from three separate databases (PubMed, JSTOR and Science Direct) related to testing the effect of EAS on PTSD outcomes in veterans. A risk of bias assessment of included studies was conducted and meta-analysis of outcomes performed when two or more studies reported the same outcomes. Other effects of EAS on veterans’ health were also discussed.

**Results:**

A total of 13 studies were identified based on our inclusion and exclusion criteria with 11 originating from the US and the remaining two from Australia and Israel. There were 344 participants amongst all of the studies with a mean age of 47 years and a male:female ratio of 19:6. Eight out of the 13 studies reported PTSD scores, as measured by either PTSD Checklist for DSM-5 (PCL-5) or PCL-Veteran/-Military versions (PCL-V/-M), and results suggested a reduction in PTSD score after EAS treatment of 22.6%. A meta-analysis confirmed that EAS favored a significantly lower PTSD score after treatment, with a mean difference of 12.46, 95% CI [9.03,15.88], *p* < 0.00001. However, only one study had low risk of bias whilst all the rest of the studies had some concerns to high risk of bias.

**Conclusions:**

EAS appeared to have a positive influence on PTSD symptoms in military veterans, significantly reducing PTSD severity scores. Other benefits of EAS may be peer support, social integration, learning new skills and bonding. However, the results of this systematic review must be interpreted with caution as almost all of the studies were of low quality. Therefore, further rigorous research is required with larger participants to be able to draw conclusions about the benefits of EAS on PTSD severity.

## Introduction

Due to continued exposure to difficult austere environments and circumstances, military personnel often develop post-traumatic stress disorder (PTSD) or suffer from moral injury [1]. When leaving service and entering civilian life, military veterans can often struggle as a result. Healthcare services offer conventional pharmacological treatments for PTSD, including selective serotonin reuptake inhibitors (SSRIs), serotonin-norepinephrine reuptake inhibitors (SNRIs), and alpha-1 adrenergic receptor antagonists. Psychotherapeutic interventions include cognitive processing therapy (CPT), eye movement desensitization and reprocessing (EMDR) along with trauma focused therapies. All of these have accumulated considerable reliable evidence for their use in the treatment of PTSD and form the basis of clinical guidelines [2]. However, dropout rates are still relatively high and suboptimal responses have been observed in clinical trials [2–5].

More recently there has been a growing interest in the use equine-assisted services (EAS) for this cohort [2]. The basic premise of EAS is to utilise horses within therapy, learning or horsemanship and is increasingly being used for trauma survivors and may be associated with reduced depression and anxiety and thus improved quality of life [6, 7]. EAS is in the early stages of development and not only lacks rigorous research, but interventions are not standardized, making it difficult to replicate studies [8]. In general, EAS includes three branches and focusses on therapy (e.g. psychotherapy involving horses (PIH)), learning (e.g., equine-assisted learning (EAL)) and horsemanship (e.g., therapeutic horseback riding (THR)). In terms of therapy, which is delivered by a licensed professional, five distinct types of therapies have been identified. These include counseling, occupational therapy, physical therapy, psychotherapy and speech-language therapy [9]. Therapy sessions can be mounted or on the ground [7] and EAS aims to improve communication skills and confidence, along with cognitive, emotional and behavioural skills [1, 10]. This is different from therapeutic sessions that involve simply learning to ride a horse or care for one. PTSD can have a profound impact on quality of life, mood and relationships and it is thought that EAS can be beneficial for all these factors [10], along with the benefits of being exposed to new environments in non-typical clinical spaces i.e. a horse-riding school. EAS brings a physical aspect to the therapy alongside conversational based approaches [1, 10].

A large proportion of military veterans are male and research suggests that they often struggle to open up; this may negatively impact engagement with conversational or psychological therapies. EAS allows much to be achieved through a bond and attachment even before a word is spoken [11]. EAT has demonstrated positive outcomes for veterans diagnosed with PTSD with 87.1% of veterans reporting very positive perceived benefits regarding relief from PTSD symptoms and 100% stated positive benefits with acquiring new or enhanced self-mediation skills [12, 13]. To understand the true benefits of EAS on PTSD symptom severity in military veterans, we aimed to perform a systematic review of existing research. We also addressed short-term outcomes; intensity and quality of the intervention; barriers to access; and outcomes (both at the time of treatment and after defined time periods).

## Methods

### Search strategy

This systematic review was not prospectively registered but was conducted in accordance with the Preferred Reporting Items for Systematic Reviews and Meta-Analyses (PRISMA) guidelines [14]. A search strategy was devised to review the existing original research in peer reviewed journals of EAT in the treatment of PTSD. Three search databases used were PubMed, JSTOR and Science Direct. MP carried out the main searches and screening with AVS assisting with the search strategy process and ZA independently confirming the screening and study selection process. Any discrepancies between the authors were resolved through discussion. Identified studies were cross-referenced, and any duplicates removed using a Microsoft Excel sheet.

### Types of studies

We initially screened for randomised controlled trials (RCTs) but since there were very few, we extended the search to include all study designs. No restrictions regarding the time to assessments or other design characteristics were applied.

### Types of comparators

Comparators included: 1) no treatment controls; 2) pre- and (3) post-EAS treatment.

### Types of outcome measures

The primary efficacy outcome was PTSD severity score using PTSD Checklist for DSM-5 (PCL-5) or the PCL-Veterans/-Military (PCL-V -M) checklists [15] in response to EAT treatment. Efficacy outcomes were analysed pre- and post-EAT treatment, without any time restrictions. The secondary effect measure included a qualitative analysis of effects on wellbeing. Other outcomes included those that made a meaningful change to PTSD severity scores were also assessed qualitatively.

The inclusion criteria were: studies including military veterans with a diagnosis of PTSD/moral injury; and delivery of equine-assisted therapy. Exclusion criteria were: children (under 18 years); civilian populations; other mental health disorders (e.g. addiction); other animals used for therapy; and non-peer reviewed articles.

### Data extraction and synthesis

After removing duplicates, titles and abstracts were screened to exclude irrelevant studies, before abstract screening and full text review. Where any discrepancies arose, this was resolved by discussion and mutual agreement between MP and AVS. Articles with the potential to be included underwent a systematic bibliography screen to identify further relevant articles.

Data on author, year, location, methods, patient demographics, EAS programme used and duration and frequency were extracted for study characteristics. The following data was extracted on outcomes: PTSD scores pre- and post-EAS treatment and clinician-administered PTSD (CAPS-5) scores. Other outcomes were qualitatively synthesized including influence of partner involvement, peer support, barriers and general outcomes to EAS programmes, and additional benefits of EAS (e.g. learning new skills and social engagement, forming relationships and bonding, exploring positive lifestyle challenges and opportunities for reflection).

### Risk of bias assessment

Risk of bias for the included studies were assessed using RoB2 for RCTs [16]; ROBINS-I [17] for interventional studies without randomisation and ROBINS-E tool [18] for the single observational study. Two authors (MP and ZA) judged the risk of bias in each study independently and any discrepancies were resolved through discussion.

### Statistical analysis

In general, PTSD measured using PCL containing more than 3 studies and was eligible for a meta-analysis. Data was pooled from all studies reporting PTSD score using any PCL checklist (either PCL-5, -V, -M), and meta-analysis conducted using RevMan 4.0 software, employing a random effects model, reporting mean difference and 95% confidence intervals (95% CI). The heterogeneity in the studies was assessed using I^2^, Chi^2^, or Tau^2^ statistic. Despite only two studies reporting CAPS-5 scores, data was pooled from the two studies to perform a meta-analysis, using the same parameters as PCL.

## Results

**Fig. 1:**
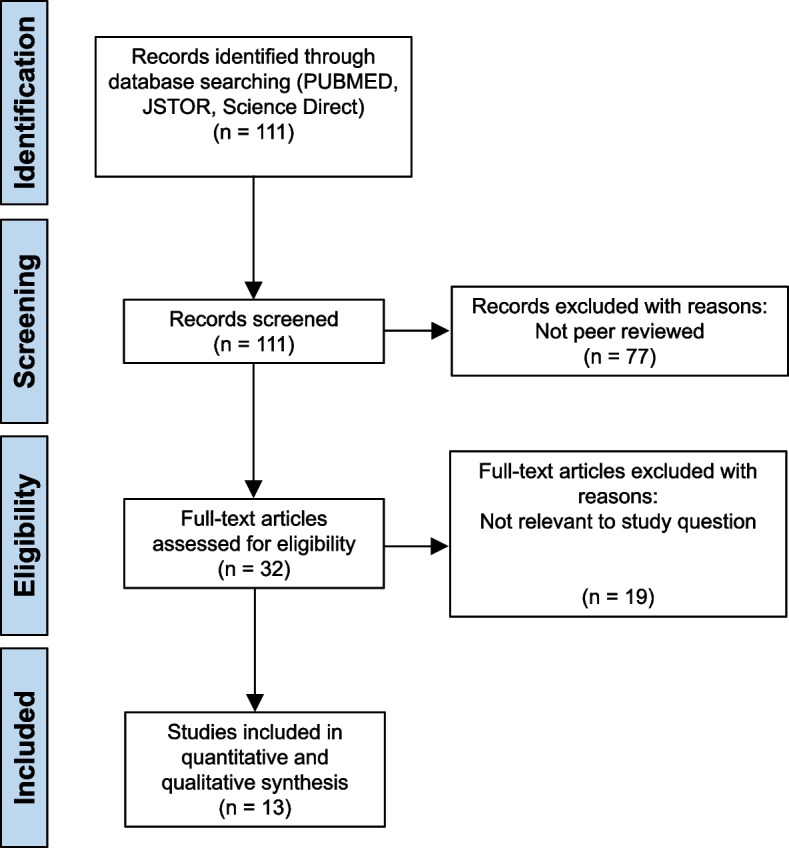
PRISMA flow chart

Searches of the three databases yielded 111 results, 12 from PubMed, 25 from JSTOR and 74 from Science Direct. From this, 77 records were excluded since they were not peer reviewed research articles. From the 32 remaining records, full text reading excluded a further 19 studies leaving 13 studies to answer our review question: is EAS beneficial for military veterans diagnosed with PTSD? Fig. 1 depicts the PRISMA flowchart that details the selection process.

### Study characteristics

The included studies were published between 2016—2023, the majority of which were from the US [19–22, 24–31] with one study from Australia [23] and one from Israel [25] (Table 1). Two studies were RCTs [19, 27] although both of these report findings from a single study, 11 were clinical studies such as evaluation studies, pilot studies or clinical open trials [20–26, 28, 29, 31] and one study was observational [30]. There were a total of 344 participants across all included studies, aged between 37–58 years, with males making up 76% of participants. The type of EAS used were a mixture of THR [19, 27, 30] or PIH [20–24, 28, 29, 31] or PIH and EAL [25, 26].

**Table 1:** Characteristics of the included studies

Study	Study origin	Type of study	n/ M:F (%)	Mean age (yrs)	Type of EAS	Summary of EAT programme	Duration and Frequency	PTSD outcome measure
Johnson et al. 2018 [19]	USA	RCT	38/ 84:16	54	THR	THR classes held at a Professional Association of Therapeutic Horsemanship accredited riding centre. Staff focused on horsemanship, tacking up, safety and grooming	One 50–60 min session per week for 6 weeks	PCL-M
Fisher et al. 2021 [20]	USA	Clinical Open Trial	63/ 63:37	50	PIH	Session co-led by licensed mental health professional and qualified equine specialist. Mental health professional to facilitate reflection, observation and direction. Psychoeducation, interaction with horses in the round pen. Grounding exercises, grooming, leading horses, team work exercises with the horse in different situations, followed by team feedback and closing circle for discussions All sessions were videotaped by researchers	One 90 min session per week for 8 weeks	PCL-5 CAPS-5
Arnon et al. 2020 [21]	USA	Clinical Open Trial	8/ 75:25	45	PIH	Session co-led by mental health professional and equine specialist. Sessions included grounding exercise, relaxation exercises, ‘join up’ activity, team work exercises and reflection opportunities	One 90 min session per week for 8 weeks	PCL-5 CAPS-5
Zhu et al. 2020 [22]	USA	Clinical Open Trial	19/ 47:53	46	PIH	Grounding exercise, teaching on EAS rationale, psychoeducation, round pen meeting with horses. Team feedback and direction	One 90 min session per week for 8 weeks	PCL-5 (no numbers presented)
Romaniuk et al. 2018 [23]	Australia	Evaluation Study	36/ 81:19	46	PIH	Session co-led by Equine Psychotherapy Institute (Australia) Program Model of Equine Assisted Learning/Psychotherapy and a registered Psychologist. Sessions involved groundwork techniques and elements of natural horsemanship, learning new skills to create social engagement. Therapeutic work to explore issues, challenges, behaviours and build awareness of responses. Opportunities to reflect. Trail walks, mindfulness and group discussions	Five day residential programme	PCL-5
Burton et al. 2019 [24]	USA	Clinical Open Trial	20/ 80:20	47	PIH	Sessions co-led by Equine Assisted Growth and Learning Association (USA) and licensed occupational therapist. Sessions included ground based exercises using horses at metaphors, leading and obstacle exercises. Pre-session discussions with an occupational therapist. Targeted psychotherapy activity, goal setting and focus on positive lifestyle choices to manage PTSD symptoms. Post-activity discussions	One session per week for 6 weeks	PCL-M
Rosing et al. 2022 [25]	Israel	Evaluation Study	12/ Not stated	41	PIH and EAL	Providers of the programme not stated. PIH and EAL (equine-assisted learning) which had elements of teaching skills; grooming and tacking up, riding activities, vaulting, mounted basketball and more. Groundwork included sharing stories and beliefs and interpretation of self through tasks with the horses. Ending with group conversation to share experiences	One 3 h session per week over a 6 month period	Qualitative measures used
Sylvia et al. 2020 [26]	USA	Evaluation Study	62/ 83:17	37	PIH and EAL	Certified team of providers including licensed clinicians, a therapeutic riding instructor and equine specialist in mental health learning. PIH along with mindfulness- based activities. Therapeutic riding, driving, EAL, groundwork, horse-human energy, herd observation and equine care	2 week intensive programme, with an EAT weekend. 3 × 2 h long sessions	PCL-5
Johnson et al. 2021 [27]	USA	RCT	20/ 90:10	52	THR	Sessions provided by a certified riding instructor and a licensed occupational therapist. Grooming, riding, exercises built upon over the six weeks	One session per week for 6 weeks	Qualitative measures used
Ferruolo 2016 [28]	USA	Pilot Programme	8/ 100:0	Not stated	PIH	Sessions provided by clinical social work graduate and 2 equine-facilitated mental health experts at an established therapeutic horse farm. Sessions consisted of 70 min of psychoeducation, 285 min of guided experimental equine activities, 315 min of group processing and personal reflection	Two day intensive option	Qualitative measures used
Malinowski et al. 2018 [29]	USA	Original Research	7/ 86:14	58	PIH	Sessions were led by a team including a licensed psychologist and a PATH licensed therapeutic riding instructor and equine specialist in mental health and learning. EAS and PIH sessions included horsemanship, obstacle course and mindfulness, horse chalking and active feeling exercises and grounding exercises mixed with association between positive feelings	5 × 1 h EAT sessions over 5 consecutive days	PCL-5
Marchland et al. 2023 [30]	USA	Observational Study	18/ Not stated	46	THR	Session adhered to PATH safety standards in a university equine facility. Intervention included two sessions of horsemanship and two sessions of trail rides lasting 45 min each. Session one and two focused on learning basic equine ground skills and mounting. Sessions three and four consisted of ride preparation, riding and debriefs	One session per week over 4 weeks. First two sessions 4 h in length, final two lasted 2 h	PCL-V
Marchland et al. 2023 [31]	USA	Evaluation Study	33/ 51.5:48.5	46	PIH	Sessions were provided by a licensed mental health clinician and an equine specialist. Activities focused on mindfulness, grooming, equine behaviour and communication, leading, group discussions (self-compassion, psychological safety, psychological resilience, ‘how would I treat a friend’, approaches to difficult and emotions and a guilt and shame discussion) and meditation	One session per week for 6 weeks lasting 90 min	PCL-V

*CAPS-5* clinician administered PCL, *EAL* equine-assisted learning, *EAS* equine-assisted Services, *PCL-5* post-traumatic stress disorder checklist (standard), *PCL-V* post-traumatic stress disorder checklist (Veteran), *PCL-M* post-traumatic stress disorder checklist (Military), *PTSD* posttraumatic stress disorder, *RCT* randomised controlled trial, *THR* therapeutic horseback riding, *N* number of participants, *M:F* male: female ratio, *PATH* Professional Association of Therapeutic Horsemanship (USA), *PIH* psychotherapy involving horses

### Risk of bias assessment

**Fig. 2:**
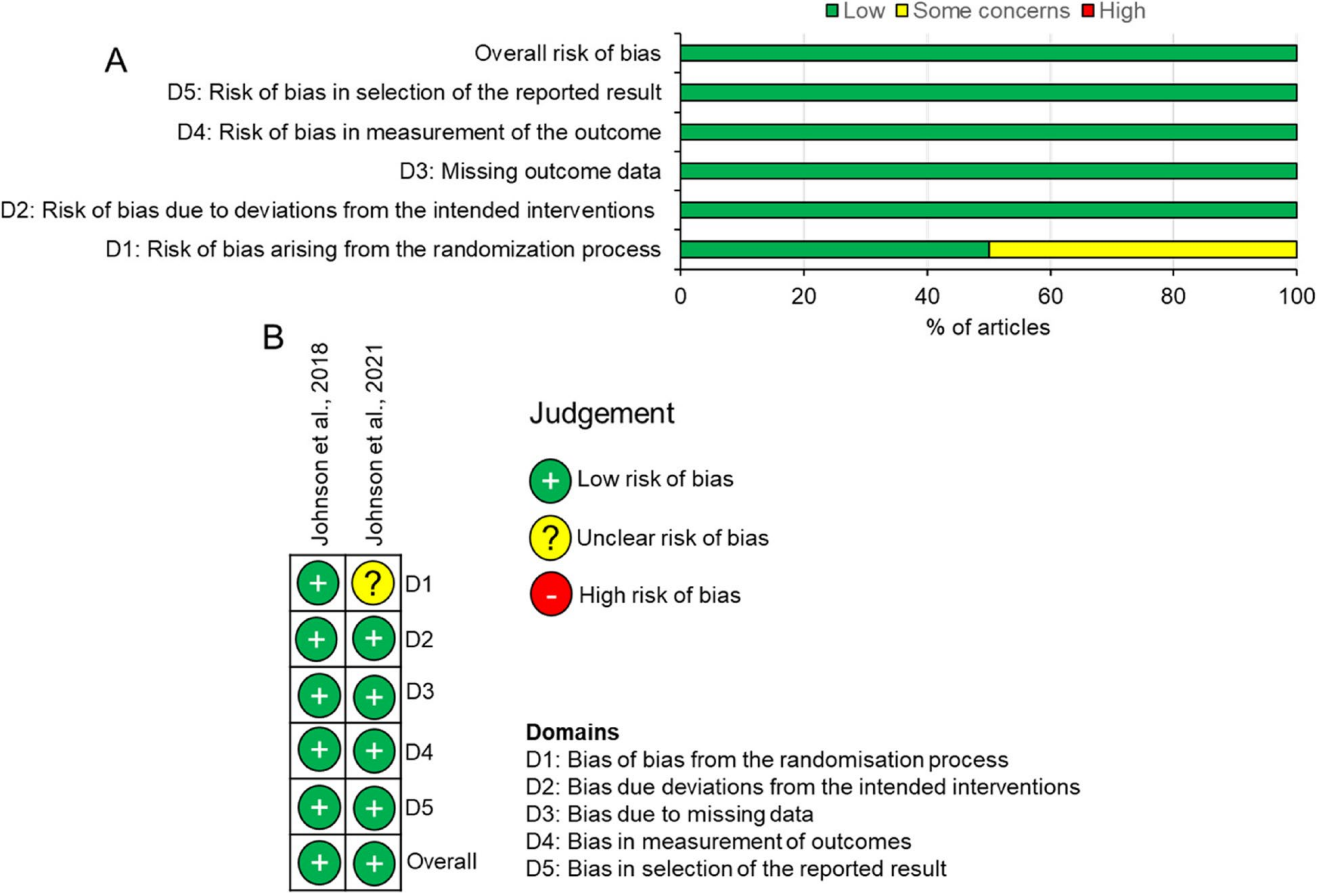
Risk of bias in included RCTs using RoB2. **A** summary graph and **B** risk of bias in individual studies

**Fig. 3:**
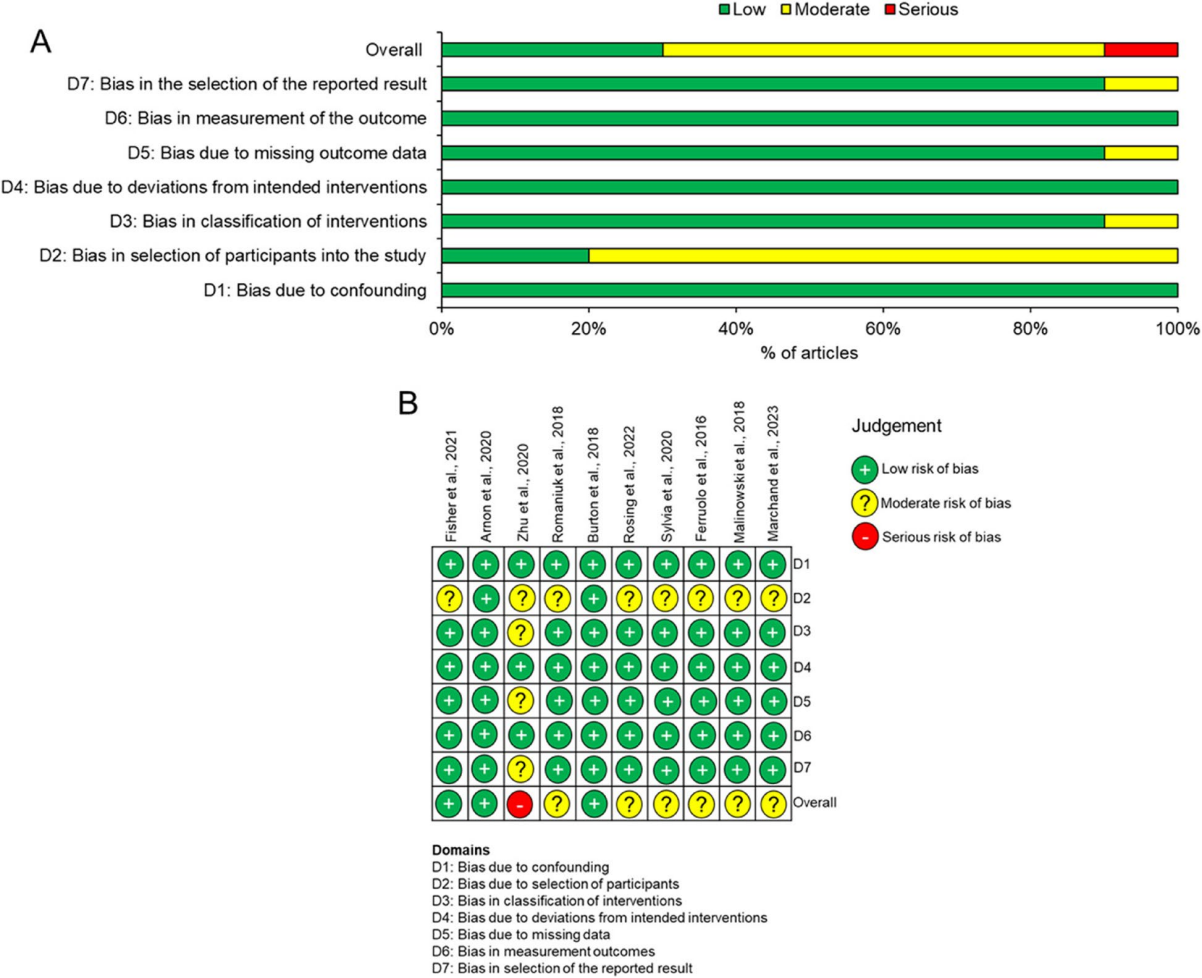
Risk of bias in included studies using ROBINS-I. **A** summary graph and **B** risk of bias in individual studies

**Fig. 4:**
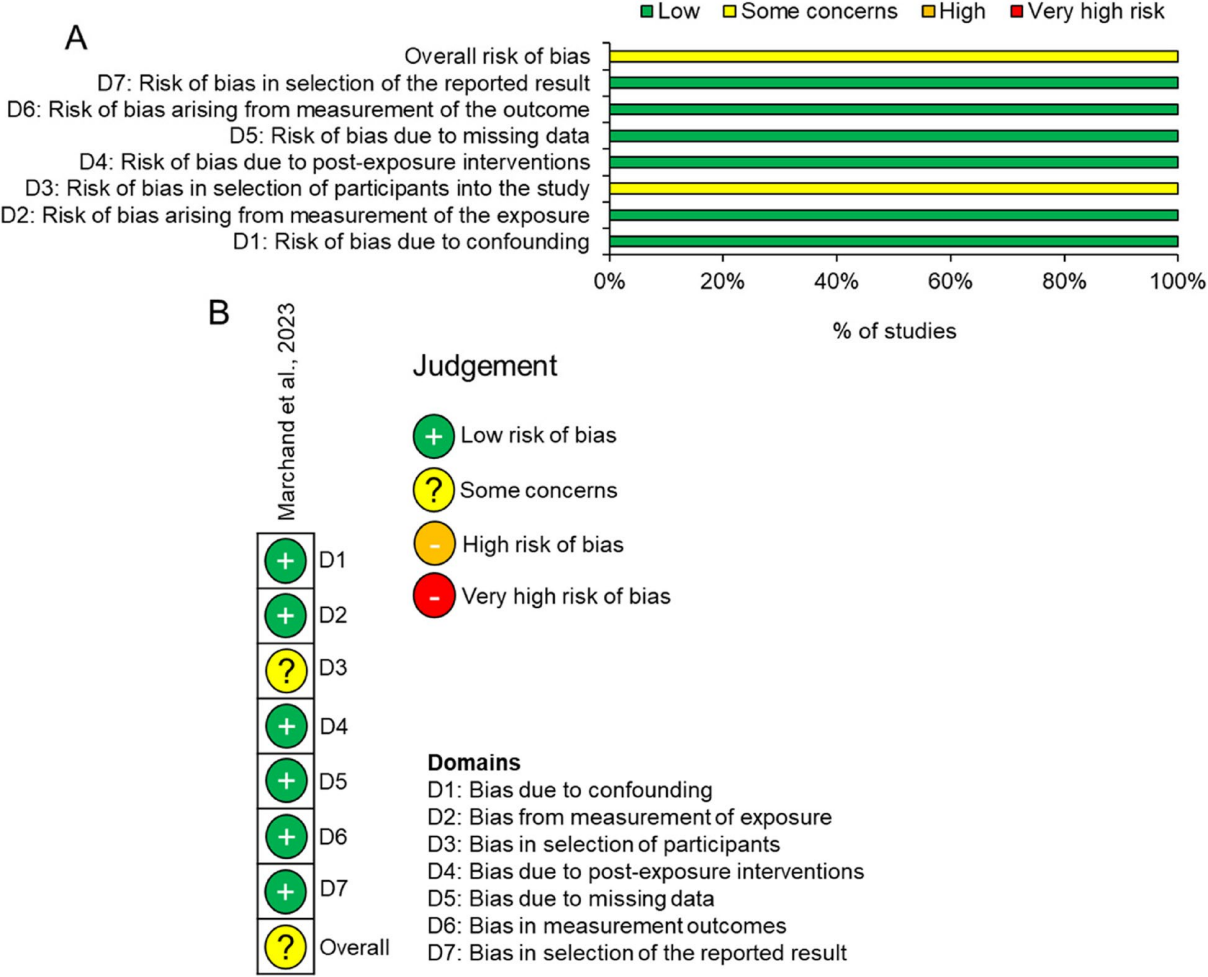
Risk of bias using ROBINS-E. **A** summary graph and **B** risk of bias in the individual study

The two included RCTs had an overall low risk of bias despite one study [15] showing some concerns in domain 1 (bias in the randomization process) (Fig. 2A and B). Of the 10 interventional studies, 30% of the articles had low risk of bias, 60% showed some concerns and 10% was judged as high risk of bias (Fig. 3A and B). In these studies, 90% had some concerns in domain 2 (bias due to selection of participants into the study) whilst domain 3, 5 and 7 had 10% of studies with some concerns (Fig. 3A and B). The overall risk of bias in the individual study was judged as moderate risk since domain 3 (bias in selection of participants) was not clear (Fig. 4A and B).

### Type of EAS treatment used

Eight of the thirteen studies included in this systematic review used PIH which involved a variety of activities led by an equine specialist along with psychotherapy provided by a licensed mental health expert [20–24, 28, 29, 31] (Table 1). Activities with horses were varied between studies but included groundwork, grooming, leading horses, teamwork, and riding. Psychotherapy was also varied and included reflection, observation, psychoeducation, goal setting and learning to make positive lifestyle choices (Table 1). When carrying out THR, the studies assigned the same horse to the participants to allow them to form a bond [19, 27, 30]. It was found that riding for an increased number of weeks led to a stronger influence in alleviating PTSD symptoms [19]. Some studies included aspect of EAL combined with PIH [25, 26], with elements of teaching skills, grooming, riding, mindfulness, and groundwork activities. Studies also varied in the duration and frequency of input, as seen in Table 1: some conducted intensive options [23, 26, 28, 29] whilst others opted for one session a week with durations that varied from 4–8 weeks [19–22, 24, 27, 30, 31] or 6 months [25] with sessions lasting from 60 min to 3 h in length (Table 1).

### Outcome measures used

Table 1 reports the different measures used in each research study and the PTSD Checklist (PCL) scores pre- and post-intervention. Out of the 13 studies, nine [19–24, 26, 29–31) used the PCL outcome measure but in different forms; PCL-5, PCL-V and PCL-M. PCL is a self-reported measure for the 20 DSM-5 PTSD symptoms, PCL-5 is the standard, PCL-V is related to veterans and PCL-M is military [19]. Other measures used include clinician-administered PTSD scale (CAPS) [20, 21], PACES [26, 31] and the PHQ-9 [31].

### PTSD symptoms

All participants in the studies were military veterans and had a PTSD diagnosis [19–31]. EAS showed short-term results (up to 6 months) in the improvement of PTSD symptoms [19–31] using varying outcome measures, but there is a lack of long-term effects [21, 23, 30, 31], and no data on follow-up after a longer time period (1 year +). Utilising the PCL outcome measure in its different forms within the studies, improvements in PTSD severity ranged from 4.02% to 50.68%, though across the included studies patients started with different baseline (pre-treatment) PCL scores and therefore a wide range of PTSD symptom severities. The mean percentage improvement in PCL score and in turn reduction in PTSD symptoms was 22.59%. However, these results are to be interpreted with caution as the design of the studies had moderate to high risk of bias, and in general studies were of low quality. The lack of standardization of interventions amongst the studies is also a cause for concern.

**Fig. 5:**
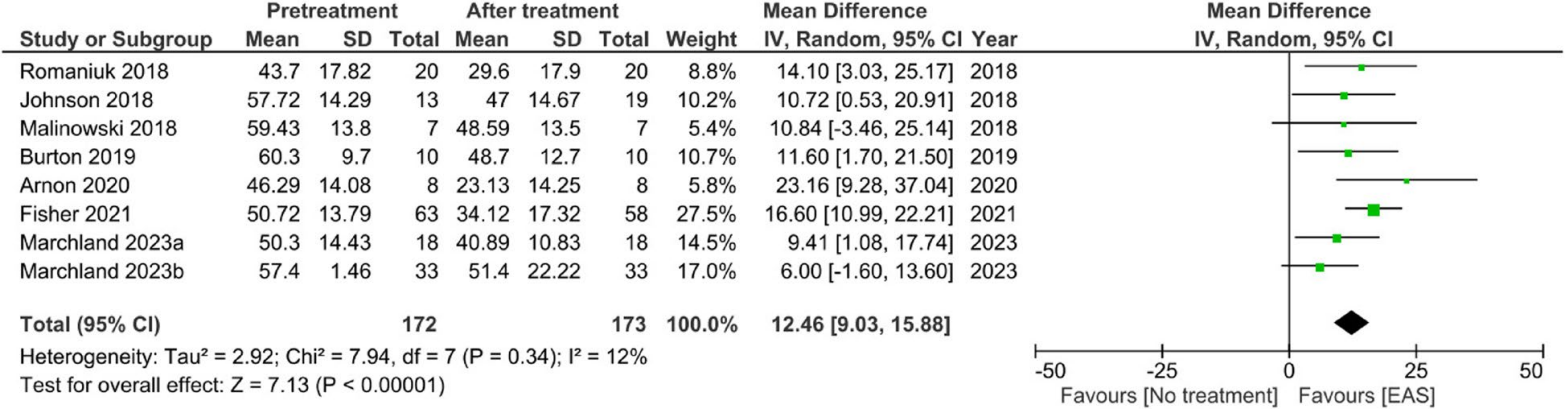
Meta-analysis for PTSD scores using PCL (PCL-5, PCL-V and PCL-M) after EAT treatment

**Fig. 6:**
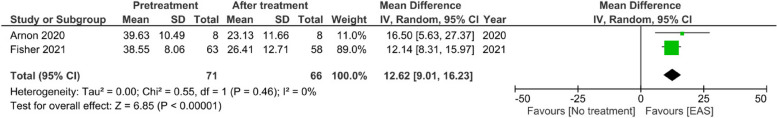
Meta-analysis for PTSD scores using CAPS-5 after EAT treatment

Despite these reservations, a meta-analysis of the studies that presented PCL scores [19–21, 23, 24, 27, 29–31], demonstrated that EAS intervention favoured a significant reduction in PTSD severity score with a mean reduction of 12.46, 95% CI [9.03,15.88], *p* < 0.00001 (Fig. 5). Despite only two studies reporting data values for the clinician administered PTSD checklist, CAPS-5, a meta-analysis showed a similar significant reduction in PTSD severity after EAS treatment, with a mean reduction of 12.62, 95% CI [9.01,16.23], *p* < 0.00001 (Fig. 6). Once again, caution must be exercised when interpreting these results due to the low quality of studies available.

### Other qualitative results for the benefits of EAS programmes

Romaniuk et al. [23] and Sylvia et al. [26] both had partner or family involvement within the EAS phase itself. It was observed that both studies had strong positive outcomes on measure of depression, stress and overall quality of life as well as greater enjoyment of both participant and family member [23, 26]. The literature also demonstrates the influence of peer support for military veterans; the social aspects of the therapy were observed to be invaluable in some studies [24]. Bringing together like-minded people allowed participants to meet new people who have had similar backgrounds and experiences, offering additional benefits to participants during the EAS programmes [25, 28].

In one study the benefits of EAS on neural systems utilising MRI to review changes, found that the limbic-basal ganglionic reward system can be influenced after undergoing EAS as a treatment for PTSD in military veterans [22]. This was proposed by the authors as supporting the benefits of EAS, as abnormal functioning of the caudate nucleus and limbic system has previously been linked to depression, substance misuse and PTSD [22[. There may also be other benefits of engagement with an EAS programme, including learning new skills and social engagement [23]; forming relationships and bonding [25, 27]; exploring positive lifestyle challenges; and opportunities for reflection [24]. However, all of these perceived benefits of EAS need to be confirmed in well controlled, high quality studies.

## Discussion

This systematic review was conducted to review existing research and evidence on the use of EAS for the treatment of PTSD in military veterans. 13 studies met the inclusion criteria and eight presented data related to PTSD scores. Out of the total number of studies utilised, 5/13 had a low risk of bias, 7/13 moderate and only 1/13 severe. Although the meta-analysis showed that EAS can improve PTSD symptoms and other associated outcome measures in the short-term, the results are to be interpreted with caution due to the low quality of studies, lack of standardization of EAS methods and baseline PTSD features of the individual. Based on the available evidence, EAS is best placed as an adjunctive strategy, in conjunction with other forms of therapy to maximise the benefits and alleviate PTSD symptoms.

This systematic review identified that EAS may offer some potential benefits in the treatment of PTSD in military veterans. For example, EAS enables participants to connect with themselves and build a bond of trust with a horse, and some evidence shows that this therapy can elicit a complete beneficial change in behaviour [27, 29]. The non-judgmental nature of horses helps foster relationships and immerses individuals in the process of bonding and ‘spiritual connection’, as observed in the studies reviewed. EAS has been previously used as a complementary therapy with other cohorts, including children with special needs and disabilities [32], patients with spinal injuries [33], and other neurological conditions.

Due to the varying nature and experience of military service, many live for years after discharge before PTSD symptoms arise or are diagnosed. PTSD has also been linked to a poor quality of life, increased use of healthcare services, family problems and addiction or substance misuse [34]. Stigma around asking for help can also create barriers to accessing appropriate professional input [35], with impact on not only the individual but their family and social support network around them. Studies in this review which utilised family and partners within treatment and therapy were shown to be more beneficial [23, 26]. This effect may also be due to shared experiences and being able to continue the work on return home. Many benefits would likely come from the group focus and peer support, along with participants feeling of being appreciated for their service [26].

The moderate risk of bias in nearly all of the studies is a cause for concern and therefore questions the validity of the results of this systematic review. Although the meta-analysis in this review demonstrated evidence that participants experienced at least some improvements in the short-term, EAS may be a beneficial complementary therapy for military veterans with PTSD. However, to draw definitive conclusions on the use of EAS will require high-quality studies to verify the results of this systematic review. Many of the included studies had small sample sizes and they allowed patients to access EAS at no cost to themselves, although large administrative burdens were likely in arranging the EAS programmes. Variables existed across the studies in terms of the veteran cohorts: how long they have served, what deployments they went on and how long they had been living with PTSD, which all play a role in how likely the reduction in PTSD symptoms would last and their amenability to therapy.

### Limitations of the included studies

The main limitations include the low quality of the studies, moderate to high risk of bias and the lack of standardization of EAS methodologies between the studies, making it difficult to make conclusive remarks about the benefits of EAS on PTSD symptoms. Therefore, the results presented in this systematic review must be interpreted with caution. For example, many of these studies had small sample sizes [24, 27, 30] and most were clinical evaluation or observational studies and did not include control groups. There was also a variety of duration of treatment, which could affect overall PTSD scores and although all participants had a diagnosis of PTSD, the causes of trauma were varied amongst the candidates having served in different areas of the military. Therefore, confounding factors including differing lengths of service, experiences of deployments and differing lengths of time in which they had been suffering with PTSD along with differing severity, may all have a bearing on the overall outcome. Furthermore, any perceived benefits of EAS on PTSD symptoms were short-term and so there is currently no evidence of any potential long-term benefits [19–31].

## Conclusion

EAS may be beneficial in PTSD symptom reduction in the short-term for military veterans but the overall quality of the studies, requires that this observation is verified in high quality, well controlled studies, with extended follow-up times.

## Data Availability

All data generated or analysed during this study are included in this published article.
